# Autogenous Healing of Early-Age Cracks in Cementitious Materials by Superabsorbent Polymers

**DOI:** 10.3390/ma13030690

**Published:** 2020-02-04

**Authors:** Geuntae Hong, Chiwon Song, Seongcheol Choi

**Affiliations:** 1 Department of Civil and Environmental Engineering, Chung-Ang University, 84 Heukseok-ro, Dongjak-gu, Seoul 06974, Korea; hgt0916@gmail.com; 2 Structural Engineering Research Institute, Korea Institute of Civil Engineering and Building Technology, 283 Goyangdae-Ro, Ilsanseo-Gu, Goyang-Si, Gyeonggi-Do 10223, Korea; schw@kict.re.kr

**Keywords:** autogenous healing, compressive strength, flow rate, superabsorbent polymer (SAP), underwater, wet/dry cycle

## Abstract

The effect of superabsorbent polymers (SAPs) on autogenous crack healing in cementitious materials with early-age cracking was investigated. SAP-containing samples exposed to wet/dry cycles showed better autogenous healing than those only exposed to wet conditions, as determined by water flow and compressive strength recovery tests. The water flow rates through cracks (380 ± 40 µm) in cement paste and cement mortar containing 1.0% SAP decreased by around 97.1–100% and 79.7–90.7%, respectively, after 14 cycles of healing compared to 1 cycle. Although the initial compressive strength decreased with SAP addition, it recovered somewhat after a 28-d healing period. Microscopy and spectroscopy results identified CaCO_3_ and/or calcium silicate hydrate (CSH) as the main healing products.

## 1. Introduction

Cracks in concrete frequently occur over the entire service life of the structure due to load- or deformation-induced stresses. Cracks degrade concrete durability by allowing penetration of, e.g., chloride ions and sulfates, which can react with the internal cementitious matrix [1,2]. Therefore, in order to increase the service life by delaying concrete deterioration, crack repair is highly necessary. In addition, as maintenance costs increase with age of the concrete structures, there is a growing interest in self-healing methods that repair cracks autogenously without external factors.

Microcracks in cementitious materials can be healed gradually in the presence of water; this process is generally referred to as autogenous healing [3,4]. Autogenous crack healing occurs via two main mechanisms: first, the cracks are filled by reaction products generated by further hydration of non-hydrated cement particles exposed to the crack surface [5]; and second, the cracks are gradually filled with precipitation of calcium carbonate (CaCO_3_) crystals [3]. Edvardsen [3] confirmed that about 50% of the concrete specimens with a crack width of 200 µm and a hydraulic gradient of 6.25 were healed completely after seven weeks of water exposure due to autogenous crack healing. Recent studies have used various approaches to increase the efficiency of autogenous healing of cementitious materials by facilitating further hydration, improving water-tightness, and reducing shrinkage; this includes the use of alternative binder materials, microfibers, expansive additives, crystalline additives, waterproofing admixtures, and internal curing agents [6,7,8,9,10]. Although autogenous healing has great potential in durability improvement, it depends on an adequate supply of water to the cracked cementitious material [4].

Superabsorbent polymers (SAPs) have been investigated for enhancing the self-healing capacity of concrete as they can rapidly swell by absorbing large amounts of water (several hundreds of times their own weight) to form a gel structure, which retains or releases the water depending on the ambient environmental conditions [11,12]. Owing to these unique characteristics, SAPs have been utilized with cementitious materials to mitigate self-desiccation, alleviate autogenous shrinkage [13,14,15], enhance mechanical properties [16,17], and improve rapid crack self-sealing [18,19,20], as well as long-term crack self-healing [21,22].

The SAP particles mixed with cementitious materials for the purpose of crack sealing and healing are observed to initially swell for a short period of time by absorbing water in fresh mixtures. Over time, the cementitious materials become dry and the SAP particles shrink by releasing the absorbed water; this leads to the formation of multiple voids in the cement matrix [18]. As the cracks are likely to occur along these voids, the SAP voids inside the cementitious materials will be exposed to the crack surface. Subsequently, when water penetrates the cracks, the dry SAP particles present in the voids on the crack surfaces can absorb this water and swell again [18]. Although the autogenous healing mechanism is the same, the healing efficiency varies depending on the crack width; this is due to the decrease in the rate of Ca^2+^ diffusion from the cement matrix to the cracks with age [23] after the surface-controlled crystal growth in the initial phase [3]. Previous studies [3,4] confirmed that the autogenous healing efficiency of cracked cementitious materials reduces without self-healing additives as the crack width increases. Yang et al. [4] suggested that the crack widths must be less than 150 µm to demonstrate a noticeable healing behavior. However, as the SAPs exposed to the crack surfaces absorb water and swell, the effective crack width is reduced [19], resulting in a significant decrease in the flow rates through cracks for a short term [18,24]. In addition, the water absorbed by the SAPs can be released to the crack surface under dry conditions; this further enhances autogenous healing for a long term [25,26]. Because the characteristics and dosage of SAPs, experimental conditions, and controlled crack width applied by each researcher are different, it is difficult to generalize the threshold crack width that can be healed by SAPs. However, it has been found that the healable crack width is increased by the incorporation of SAPs [17]. Therefore, in order to quantify the crack width that can be healed through SAPs, it is necessary to accumulate experimental data for various crack widths.

Furthermore, the majority of the cast-in-situ concretes are exposed to an atmosphere wherein the temperature and relative humidity (RH) are continuously changing. Such conditions result in the formation of a large number of early-age cracks within several days after casting due to the volume changes induced by chemical, autogenous, and drying shrinkages, which are a result of temperature and RH cycles [17,27]; this leads to a significant degradation in the long-term concrete performance. Hence, controlling early-age cracking is critical to ensure long-term stability of concrete structures, which are ubiquitous in modern society, and avoid the cost and potential material and human damage of structural damage or collapse. Although previous studies have already demonstrated that autogenous healing of cracks in cementitious materials is enhanced by SAPs [25,26], the evaluations of the healing performance for the relatively large cracks (≥300 µm) occurring at early ages are lacking. In other words, numerous studies have addressed the autogenous healing of cracks that occurs within days or even several months. However, the majority of the research has been limited to micro-cracks (≤150 µm). Although the widths of early-age cracks are generally less than 300 µm approximately, larger cracks may occur depending on the exposure conditions of the concrete. Therefore, an analysis is necessitated for studying the influence of SAP on the healing of larger cracks to achieve a practical application of the self-healing concrete using SAPs.

Previous studies [18,28] have investigated the effect of SAPs on cracked cementitious materials in terms of the rapid self-sealing and autogenous healing. However, the results of the former study [18] are limited to the first self-sealing process, which occurs due to the swelling of SAPs by the ingress of water through cracks; therefore, this is not sufficient to predict the long-term crack healing. In addition, the latter study [28] confirmed that the autogenous healing efficiency of cementitious materials with early-age cracking was improved by the SAPs that have been used in this study under cyclic wet/dry conditions; this was established by employing a capillary water absorption test. However, considering that the main goal of crack healing is to limit the water penetration from the external environment, there is insufficient evidence to confirm whether the reduction of water ingress through cracks was entirely due to the SAPs. With regard to crack healing, the accuracy of the capillary water absorption test is debatable. In other words, partially healed cracks can comprise a smaller crack width, and therefore exhibit higher capillary forces; this can cause more water absorption than that observed in unhealed cracks. Furthermore, the recovery of the mechanical performance of the cementitious materials after autogenous crack healing by SAPs has not been investigated, and the experimental variables utilized (such as number of cycles and crack widths) have also been limited. Moreover, the abovementioned experiments were only performed using SAP-containing cement pastes.

Consequently, the aim of this study was to further analyze the effect of SAPs on the autogenous healing of early-age cracks in cementitious materials by considering more variables. Specimens containing either cement paste or cement mortar were prepared with various SAP dosages and particle sizes. The chemical and autogenous shrinkages of cementitious materials generally occur within 7 d to a considerable degree after casting [29]; further, a drying shrinkage occurs significantly within a few days after the materials are exposed to the atmosphere [30]. Therefore, we ascertained that the risk of early-age cracking would be the maximum within the period of 7 d, resulting in the formation of cracks at the 7 d for all specimens. In particular, for the reasons mentioned above, relatively large crack widths (≥300 µm) were induced in the water flow test specimens; such cracks have not generally been applied in existing studies. Subsequently, we exposed the cracked specimens to wet and wet/dry cyclic conditions to stimulate healing. Water flow tests and compression tests were then carried out to quantitatively evaluate the autogenous crack healing effect. The water flow test investigates the amount of water runoff through the cracks. The driving force utilized in this test is water pressure, which can overcome the limitations associated with the accuracy of the capillary water absorption test that was conducted in a previous study [28] as mentioned above. In addition, microscopy was used to compare the crack width before and after healing and to study the healing products. We believe that the results obtained via this systematic study will provide a deeper understanding of the self-healing enhancement of SAP in cementitious materials.

## 2. Materials and Methods

### 2.1. Materials

Type-I ordinary Portland cement (OPC; CEM I, 42.5 N, SsangYong Cement, Seoul, Korea) complying with ISO 679, with a Blaine fineness of 3499 cm^2^/g and a density of 3.13 g/cm^3^ was used in this study. Standard sand with a SiO_2_ content of >98% and polycarboxylate superplasticizer (SP8HU, BASF, Ludwigshafen, Germany) were used in the mortar mixtures. In this experiment, standard sand with a relatively small particle size of 0.59 mm or less was used. This sand was intended to manufacture the mortar specimens with uniform characteristics by minimizing the variations in the physical and chemical properties, including the particle size distribution. Table 1 shows the chemical composition of the OPC used in this study.

The cementitious mixtures used in the tests are given in Table 2. The water–cement ratio (w/c) and sand–cement (s/c) ratios were 0.35 and 1.7, respectively. Previous studies showed that excellent self-sealing of cracks was achieved when 0.5–1.5% of SAPs by weight of cement (hereafter referred to simply as wt.%) were incorporated [18,19,20]. In addition, the 7-d strength of the cement paste mixture with 1.5 wt.% of SAPs was about 50% of that of the reference mixture without SAPs [28]. This study aimed to evaluate the recovery of the mechanical properties as a method for evaluating autogenous crack healing. Therefore, 0.5 wt.% and 1.0 wt.% SAPs were used considering the self-healing efficiency and strength reduction ratio of SAP-containing cementitious mixtures. The absorption capacities of the SAPs reduce the flowability of the cementitious materials. Therefore, the amount of additional water absorbed into the SAPs was determined in order to attain the same flowability of the SAP-containing mixture as that observed in a mixture without SAPs. The addition of 10 g of water per 1 g of SAPs resulted in the same flow of 180 mm for both the mixtures [28]. Further, the determined additional water amount is presented in Table 2.

**Figure 1:**
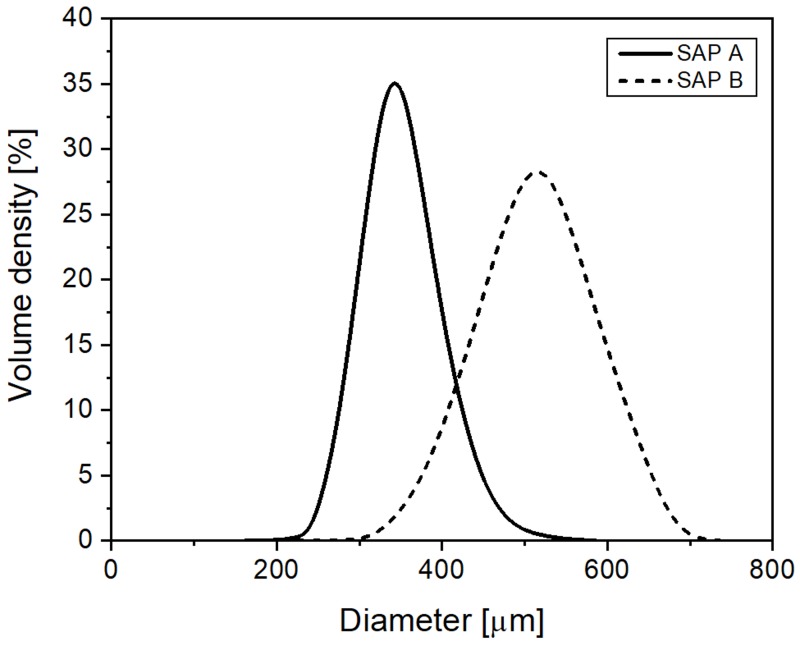
Particle size distributions of dry SAPs used in the test.

**Figure 2:**
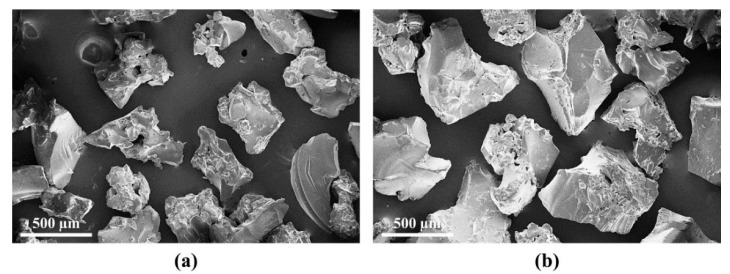
SEM micrographs of the irregular SAP particles of (**a**) SAP A and (**b**) SAP B.

The SAP material used here was a cross-linked sodium salt polyacrylate in the form of a white power produced by bulk polymerization. In order to analyze the effect of SAP particle size on autogenous healing, the SAP powder was classified using sieves (following ASTM E 11) into two particle-size ranges, 300–355 µm and 425–600 µm, which are henceforth referred to as SAP A and B, respectively. The size and type of SAP are ideal to promote the crack self-sealing [20] and autogenous healing [28]. Figure 1 shows the particle-size distribution of dry SAP powders measured using a particle size analyzer (Mastersizer 3000, Malvern Instruments, Worcestershire, UK). Approximately 10,000 SAP particles were measured, and the mean particle size of the dry SAPs was ~343 µm for SAP A, and ~522 µm for SAP B. Figure 2 shows scanning electron microscopy (SEM; S-3400 N, Hitachi, Tokyo, Japan) images of the SAP samples. Both SAP A and B particles were irregular, with similar surface textures, while the larger average particle size of SAP B is clear from the images.

To evaluate the swelling characteristics of the SAPs, the absorption capacity was measured using the tea-bag method [31] by calculating the difference between the weights of the SAPs in the initial dry state and in the swollen state after absorbing the solution. The tea-bag method is the most common and easy way of measuring the absorption capacity of small amounts of SAPs [32]. The absorption capacity was measured after 20 min of exposure of SAPs to the solution, because it reaches the maximum within approximately 10 min in all solutions. The absorption capacities (g/g) of SAP A and B were about 138 and 141 in distilled water (DW, pH: 7), 114 and 112 in tap water (TW, pH: 7.5), and 30 and 32 in filtered cement pore solution (FCPS, pH: 12.8), respectively. These differences occur because the swelling ratio of SAP depends on the pH, ion species, ion concentration, and other properties of the solution [18,33]. In particular, the cement pore solution contains a variety of ions (e.g., Ca^2+^, K^+^, Na^+^, OH^–^), which results in solutions with a high ion concentration and pH [34], and hence, decreased swelling of the SAPs [35,36]. In addition, the swelling of SAPs is limited by the complexation formed by the bond between the Ca^2+^ in the cement pore solution and the carboxyl group of the SAPs [20,37]. The difference in absorption capacities of SAP A and B was attributed to experimental uncertainty and deemed negligible. In addition, the particle sizes of the SAPs in the swollen state were measured 20 min after these SAPs were immersed in each solution, as in the case of the absorption capacity measurement; the measurements were carried out by using a particle size analyzer. The mean particle diameters of SAP A and B were approximately 1509 µm and 1772 µm in DW, 1139 µm and 1303 µm in TW, and 467 µm and 627 µm in FCPS, respectively. Although the results are less reliable because the particle size analyzer assumes that the SAP particles are spherical, the tendency of the degree of swelling according to the solution is consistent with the results of the absorption capacity.

### 2.2. Samples

The reference mixtures without SAPs were mixed according to ISO 679. The cement paste (CP) and cement mortar (CM) mixtures incorporating SAPs were mixed according to the ISO 679 procedure after dry mixing both cement and SAPs for 1 min to achieve homogeneous distribution of the SAP particles. For the CM mixtures, the superplasticizer was pre-mixed with the total water (mixing water and additional water to be absorbed by the SAPs).

**Figure 3:**
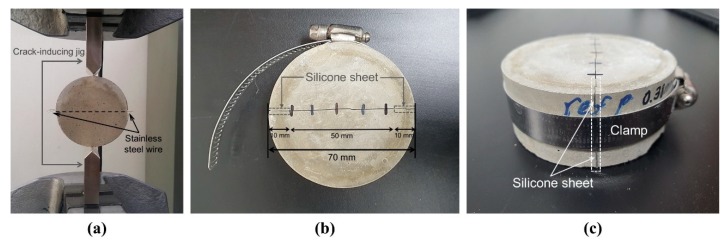
Preparation of the water flow test specimen. (**a**) MTS 810 with crack-inducing jigs to induce through-crack, (**b**) crack width control using silicone sheets, and (**c**) the assembled cracked specimen.

To evaluate the compressive strength recovery, 12 cubic specimens of 50 × 50 × 50 mm^3^ were prepared for each mixture, while for the water flow test, 12 cylindrical specimens with a diameter of 70 mm and a height of 30 mm were prepared for each mixture (Table 2). As the autogenous healing of early-age cracks was of interest, both specimen types were cast into the molds and stored for 7 d under the standard curing conditions, which comprise the constant temperature of 23 ± 1 °C and the relative humidity of 100% RH. Figure 3 shows the preparation procedure of the water flow test specimens. As shown in Figure 3a, a 0.3-mm-diameter stainless steel wire was placed perpendicular to the expected crack direction inside each specimen to prevent loss of SAPs and damage to the crack surface due to splitting of the specimen during crack formation [20]. After curing, through-cracks were induced at the centers of all the specimens by tensile splitting with a universal testing machine (MTS 810, MTS Systems Corporation, Minneapolis, MN, USA) and the cracked specimens were dried at constant temperature and RH conditions (20 ± 1 °C, 50 ± 1% RH) for 72 h in order to simulate the state of dried SAPs after exposure to the crack surface in a real environment. After drying, the crack widths of the 12 specimens for each mixture were controlled with a silicone sheet with a thickness of 380 µm. Silicone sheets were inserted at both ends of the crack surface for all specimens, and the side surface of each specimen was tightened with a clamp (Figure 3b,c). Five points were selected at equal intervals in the cracks on the top and bottom surfaces of the specimens, and the surface crack width was measured twice for each measurement point so that the mean surface crack width of each specimen could be calculated. The details of the sample preparation and crack width measurements for the water flow test are presented in our previous study [20]. Although the crack width was controlled by a silicone sheet of a certain thickness, the mean surface crack widths for each specimen measured by optical microscopy were in the range of 380 ± 40 µm. It has been established before that autogenous healing can occur in microcracks [3,4]; however, our previous study [19] indicated that the effective crack width can be significantly reduced by the swollen SAPs. Therefore, the crack width used here was determined based on the assumption that the cracks could be sealed and healed by the SAPs used in this study; this was achieved by taking into account their particle sizes and swelling ratios.

### 2.3. Test Methods

#### 2.3.1. Water Flow Test and Microscopy

**Figure 4:**
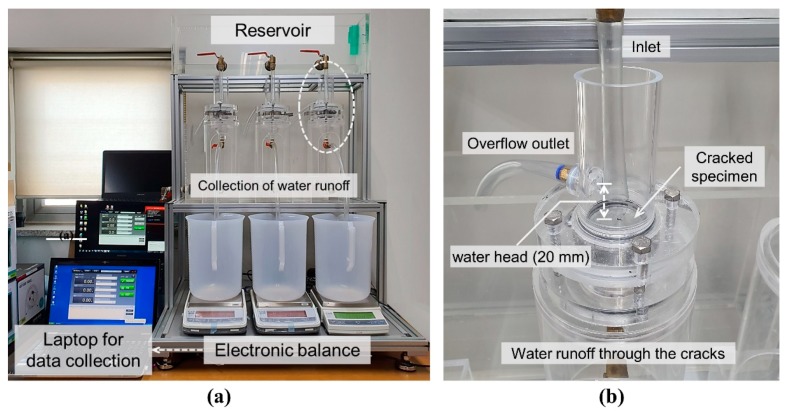
(**a**) Experimental setup for the water flow test and (**b**) an enlarged photograph of the white dotted circle in left figure.

**Figure 5:**
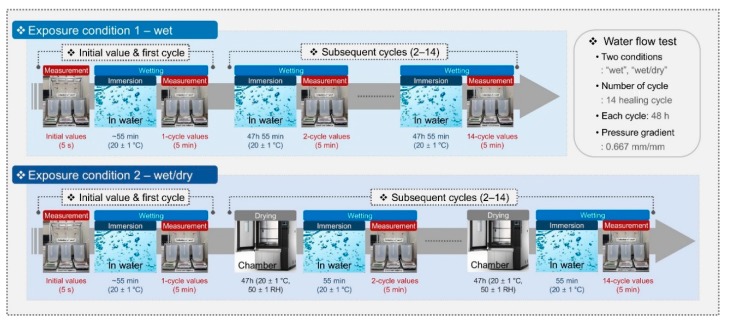
Exposure conditions for measuring the flow rate through cracks during the water flow test.

Figure 4 shows the experimental setup for the water flow test. The water flow test was carried out for 28 d in a controlled environment (20 ± 1 °C, 50 ± 1% RH) on the assembled cracked specimens (Figure 3c) after 7 d of standard curing. During the measurement, the water pressure head at the water ingress was provided for the cracked specimens with a height of 30 mm; further, it was maintained uniformly at 20 mm, i.e., the pressure gradient was 0.667 mm/mm, as shown in Figure 4. Distilled water was used for the ingress of water, and the amount of water runoff through the cracks was measured at 5-s intervals over time by using electronic balances and laptops. In order to analyze the effect of the exposure conditions on the autogenous healing after cracking, two different conditions (1 and 2) were imposed on the cracked specimens during the healing period, as shown in Figure 5. The water runoff through cracks per unit time was evaluated in terms of the water flow rate, which in turn was expressed in terms of the water permeability coefficient in existing studies [3,4,5]. The flow rate was measured repeatedly at intervals of 48 h according to the cycle. Condition 1 included 14 healing cycles under water (“wet” samples), wherein each cycle consisted of continuous wetting for 48 h (20 ± 1 °C). Condition 2 consisted of 14 healing cycles as well, but under wet/dry conditions (“wet/dry” samples), wherein each cycle consisted of wetting for 1 h (20 ± 1 °C) and drying for 47 h in a chamber with a controlled environment (20 ± 1 °C, 50 ± 1% RH).

For the initial measurement and the first cycle, we measured the mean flow rate through the cracks for the first 5 s (taken as the initial values), which was followed by remeasuring the mean flow rate for 5 min (taken as the first cycle values) after immersion in water for 55 min. For the subsequent cycles (2–14), we measured the mean flow rate for 5 min per cycle at 48 h intervals (taken as each cycle values) for the wet specimens; whereas, for the wet/dry specimens, we measured the mean flow rate over the last 5 min after immersion in water for 55 min, during the wetting in each cycle (taken as each cycle values). Even though the surface crack widths of the crack-controlled specimens were nominally the same, water permeability varied due to different internal crack geometries for each specimen [38]. Therefore, in order to more accurately analyze the healing efficiency of SAPs for the same crack width, the initial flow rate during the first 5 s due to the first ingress of water after cracking was measured for all specimens. Then, specimens with similar initial values were selected for further measurements. It is interesting to note that the measured crack widths of the specimens with similar initial flow rates were also comparable. Consequently, six specimens from the 12 prepared were selected for each mixture, while the selected specimens were classified into three groups depending on the magnitude of the initial flow rate. Then, three of these specimens were tested under exposure condition 1, while the remaining three were tested under exposure condition 2. After each cycle, the specimens were removed from the testing equipment and repeatedly exposed to the curing conditions (wet or wet/dry cyclic conditions). Finally, optical microscopy was used to evaluate surface crack closure and confirm whether the healing products were formed and/or the crack width decreased after the healing cycles. Surface crack images were captured before and after a 28-d water flow test at the same crack location.

#### 2.3.2. Test on the Recovery of Compressive Strength

Compressive strength recovery tests were carried out based on a previous study [39]. The compressive strengths of the cubic specimens were measured following ASTM C 109 immediately after 7 d of standard curing, immediately after pre-damage, and after the healing period. Compressive strength measurement and pre-cracking were carried out under load control at a load rate of 900 N/s using a universal testing machine (MTS 810, MTS Systems Corporation, Minneapolis, MN, USA). The experimental program consisted of four steps:

1. Three specimens were tested to evaluate the initial 7-day compressive strength (*f_c_*_(7)_);
2. The remaining nine specimens were pre-damaged using a stress equal to 80% of *f_c_*_(7)_ to obtain a preset damage level;
3. Three pre-damaged specimens were re-tested up to failure immediately after pre-cracking, obtaining the compressive strength (f ^*^_c__(7)_);
4. The remaining six pre-damaged specimens were re-tested up to the maximum achievable compressive strength after curing under the two different healing conditions, obtaining (f ^*^_c__(7+28)_).

Here f ^*^_c__(7+28, *h.p.w*)_ and f ^*^_c__(7+28, *h.p.w/d*)_ are the compressive strengths after healing period under wet and wet/dry conditions, respectively.

#### 2.3.3. Characterization of the Healing Products

Scanning electron microscopy (SEM; S-3400 N, Hitachi, Tokyo, Japan) and Fourier transform infrared spectroscopy (FTIR; TENSOR 27, Bruker, Karlsruhe, Germany) tests were performed to analyze the morphology and chemical composition of the healing products promoted by the SAPs. SEM analysis with energy dispersive X-ray spectroscopy (EDS) was carried out in secondary-electron mode on fragments collected from internal cracks of the water flow test specimens after completion of the water flow test for 28 d. For this purpose, the clamp was dismantled from the specimen, and then the specimen was carefully separated into two parts along the crack, and the samples including the internal crack surfaces (after 28 d under wet or wet/dry cycles) were taken. It was revealed that the formed healing products made such differences that the collected samples at the internal crack surfaces were whiter than those at the internal cement matrix. Prior to SEM-EDS analysis, the collected samples were coated with a thin platinum layer using a plasma magnetron sputter coater after drying and vacuum conditioning. For FT-IR analysis, the white crystalline material generated on the external surface cracks of the SAP-containing specimens after completion of the water flow test was carefully removed from the cement matrix and pulverized. These powders were kept under vacuum until measurement so that such further reactions as carbonation and complexation formation could be prevented.

## 3. Experimental Results and Discussion

### 3.1. Water Flow through Cracks

**Figure 6:**
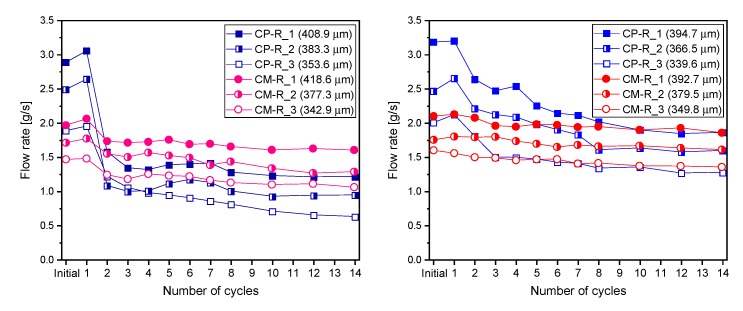

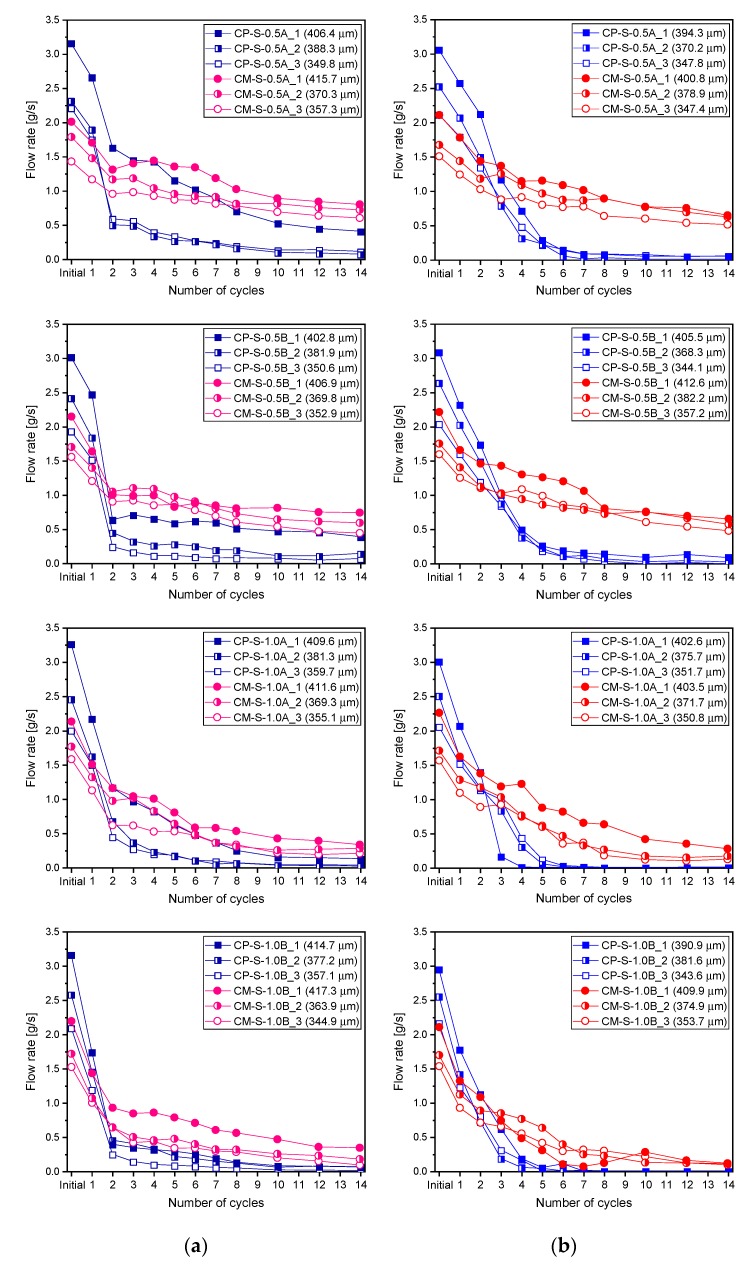
Flow rate as a function of the cycle number for cracked specimens with and without SAPs under either (**a**) wet or (**b**) wet/dry healing conditions.

Figure 6 shows the mean flow rate through the cracks per unit time as a function of cycle number. The initial flow rate and its changes were measured over the cycles, depending on the exposure conditions (Figure 5) during the test.

The initial flow rates of all specimens increased with increasing crack width. For similar crack widths, the flow rate was higher for CP specimens than CM ones. The initial flow rates were 1.886–3.258 g/s and 1.426–2.264 g/s for the CP specimens with a crack width of 340–415 µm and CM specimens with a crack width of 343–419 µm, respectively. The water flow through cracked cementitious materials is strongly influenced by the crack geometry (surface roughness, tortuosity, porosity, width, etc.) [19,38]. It can be also affected by micro-cracks that are generated close to a single induced crack. Although through-cracks were induced for all water flow test specimens using the same technique, the degree of tortuosity of the crack surfaces of CP and CM specimens was different, as shown later. In particular, the degree of crack tortuosity of the CM specimens was higher than that of the CP specimens, attributed to the greater inhomogeneity of the CM mixtures.

Interestingly, the trends in the flow rate from the initial stage to the first cycle were different for the cracked CP and CM specimens with and without SAP. In the case of the CP–R and CM–R specimens without SAP, the flow rates in the first cycle were almost the same or slightly higher than the initial values. As both of these values were measured within the first hour of water ingress, it is unlikely that crack self-healing occurred. Therefore, the difference was attributed to the effect of air bubbles [22] on the internal crack surfaces, which were present during the initial measurement, but were removed during immersion in water, resulting in the slightly higher flow rate measured for the first cycle. On the other hand, in the case of the CP–S and CM–S specimens with SAP, rapid crack self-sealing due to the swelling of SAP particles by water absorption [18,19,20] overcame the effect of the air bubbles, resulting in a significant decrease in the mean flow rates during the first cycle compared to the initial stage.

Figure 6 shows that the mean flow rates of all cracked specimens gradually decreased over the healing period, where the greatest decrease was observed between the 1st and 2nd cycles in most cases. This was attributed to autogenous crack healing by further hydration of the un-hydrated cement particles exposed to the crack surface [22,28], despite a relatively short period of 48 h between the cycles. In addition, the flow rate reduction between the 1st and 2nd cycles was greater for wet healing conditions than for the wet/dry conditions. This was probably due to a higher degree of autogenous healing in the sample exposed to wet conditions by occurring further hydration attributed to the continuous supply of water. Furthermore, the flow rate reduction ratios of all cracked specimens were larger in the early cycles than in subsequent cycles, where greater reductions were observed for the CP specimens than for the CM specimens due to differences in the rate of Ca^2+^ diffusion from the cement matrix to the cracks with age [23] and differences in the amount of un-hydrated cement particles [28].

Overall, the mean flow rates of specimens containing SAPs decreased more compared to those of specimens without SAPs during the testing period. For the CP–R and CM–R specimens, the flow rate reduction significantly decreased after the 3rd cycle, even though the differences were dependent on mixture, exposure condition, and crack width. The flow rates did not converge towards zero until the end of the experiment. However, the flow rates of the CP–S and CM–S specimens continuously decreased, even after the 3rd cycle. In the case of the CP–S–0.5 specimens with cracks below a crack width of about 380 µm, the flow rates reached almost zero by the end of the experiment, with little difference in the exposure conditions and SAP particle sizes. Greater flow rate reductions were observed with increasing SAP dosage; in particular, the flow rates of most CP–S–1.0 specimens converged to zero at the end of the experiment, regardless of the exposure conditions, SAP particle size, and crack width.

**Figure 7:**
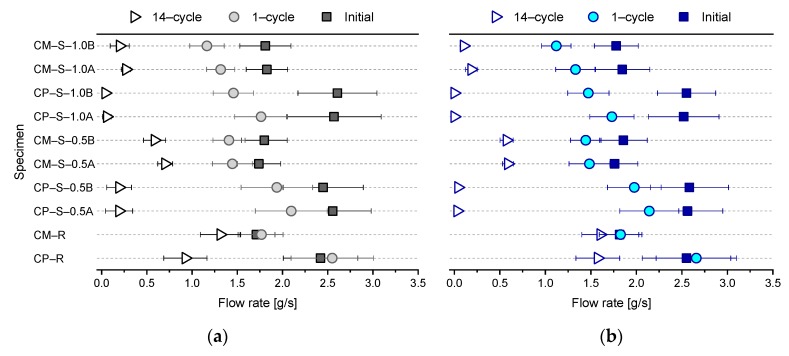
Mean and standard deviation of the flow rates through the water flow test specimens at the initial stage and after the 1st and 14th cycles under (**a**) wet and (**b**) wet/dry conditions.

**Figure 8:**
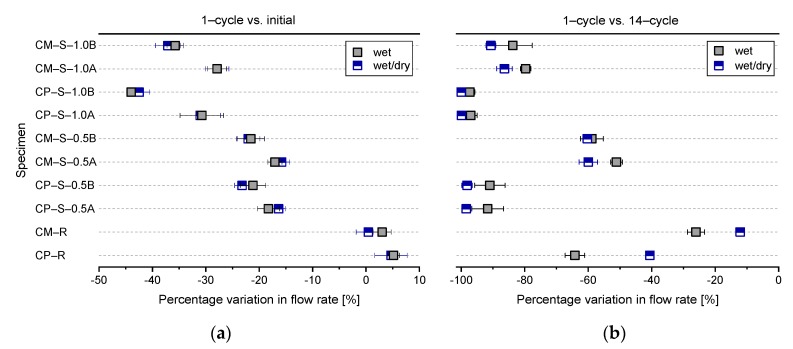
Mean and standard deviation of the percentage variation in the flow rate for the water flow test specimens comparing the values for the (**a**) 1st cycle with the initial values, and (**b**) 14th and 1st cycles.

Figure 7 shows the mean and standard deviation of the flow rates (from Figure 6) as a function of the crack width at the initial stage, 1st cycle, and 14th cycle of the water flow tests for each mixture. Figure 8 shows the percentage variation in flow rate, comparing the values shown in Figure 7 for the 1st cycle with the initial values, and comparing the values from the 14th cycle with those from the 1st cycle. The mean flow rates through the CP–R and CM–R specimens increased slightly (up to +5.1%) in the 1st cycle compared to the initial stage, while the corresponding values for the CP–S and CM–S specimens slightly decreased, with a variation in the range of −15.8–23.2% for the CP–S–0.5 and CM–S–0.5 specimens, and −30.7–43.9% for the CP–S–1.0 and CM–S–1.0 specimens. The absolute values of the percentage variation increased with increasing SAP dosage due to enhanced rapid crack self-sealing [18,19,20]. In addition, as shown in Figure 8a, SAP B with the larger particle size showed better rapid crack self-sealing performance under the controlled crack width conditions, which may be attributed to the larger particles being able to swell sufficiently to fill voids, including cracks [18], while SAP A could not swell as much. In addition, due to the differences in crack geometry according to SAP type and SAP shape, the irregular SAP particles used here may exhibit different swelling behavior compared to that of spherical SAPs [18]. Therefore, further studies are required to analyze the swelling behavior of irregularly shaped SAPs in cracks, considering more variables via image analysis, such as cryofracture SEM and X-ray CT analysis.

Furthermore, although the initial flow rates were similar, the mean flow rates over 14 cycles for the CP–S and CM–S specimens were significantly lower than those of the CP–R and CM–R specimens (Figure 7**)**. For the CP–R and CM–R specimens, the percentage variations comparing the 14th and 1st cycles were about −40.6–64.2% and −12.1–26.1%, respectively, although there were differences depending on the exposure conditions during the healing period (Figure 8). On the other hand, the corresponding values for the CP–S–0.5 and CM–S–0.5 specimens decreased about 90.9–98.4% and 51.1–60.3%, respectively, and that of the CP–S–1.0 and CM–S–1.0 specimens decreased about 97.1–100% and 79.7–90.7%, respectively (Figure 8). Overall, it was confirmed that the flow of water through the cracks was effectively blocked by SAPs; however, these results were observed only in the low-pressure water flow test. Furthermore, because the water flow tests were conducted in a limited number of cycles, it was difficult to conclude whether the flow rate continuously decreased or converged when the test was repeated after 14 cycles. Therefore, further study is needed to evaluate the variation in the long-term flow rate with various water pressures.

In summary, as shown in Figure 6, Figure 7 and Figure 8, some autogenous crack healing occurred in the cracked cementitious materials without SAPs due to hydration of un-hydrated cement particles, although the crack-healing efficiency was greatly improved when SAPs were included. In particular, the autogenous crack-healing efficiency of CP–R and CM–R specimens was improved when exposed to wet compared to wet/dry cycling, attributed to the continuous supply of water that facilitated the formation of healing products. However, the autogenous crack healing efficiency of CP–S and CM–S specimens was improved when exposed to wet/dry compared to wet cycles. These differences are discussed in more detail in Section 3.2. Additionally, the exposure conditions after cracking applied in this study could be relatively favorable for crack healing, and the results may vary if the exposure conditions change.

### 3.2. Surface Crack Closure Test

**Figure 9:**
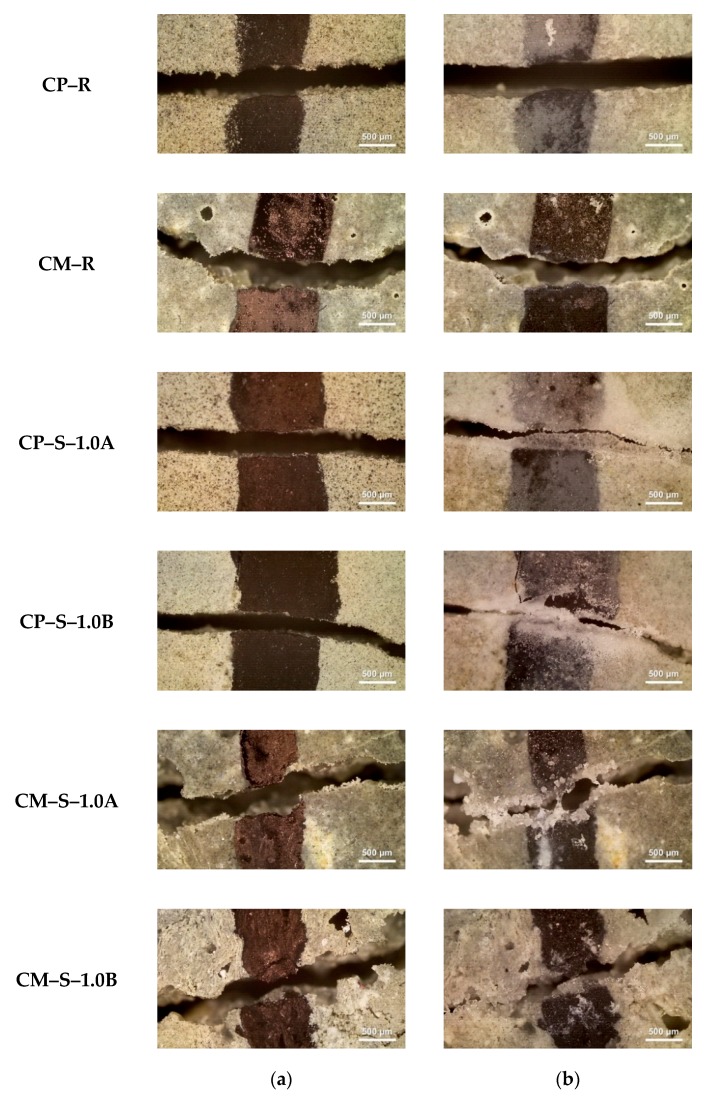
Photomicrographs for external surface cracks of specimens obtained using optical microscopy. The micrographs highlight (**a**) right after crack occurrence and (**b**) after healing period under wet conditions (28 d).

**Figure 10:**
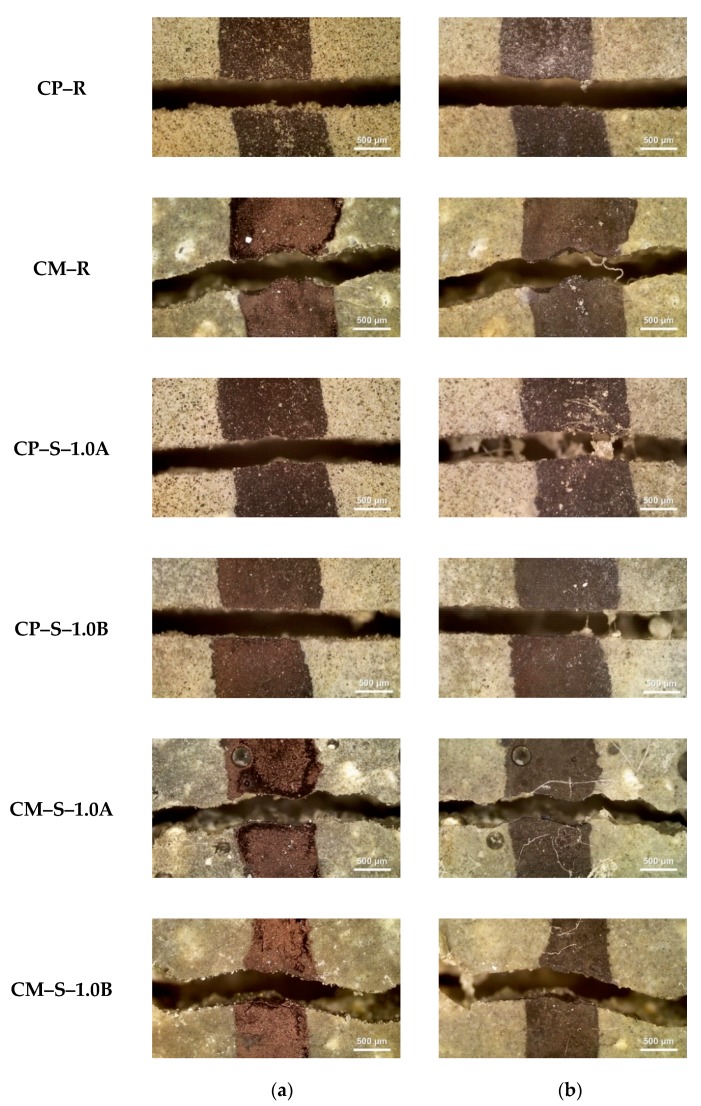
Photomicrographs for external surface cracks of specimens obtained using optical microscopy. The micrographs highlight (**a**) right after crack occurrence and (**b**) after healing period under wet/dry conditions (28 d).

Figure 9 and Figure 10 show representative optical micrographs of the external surface cracks on the water flow test specimens before and after healing. In the case of the CP–R and CM–R specimens without SAPs, regardless of exposure conditions, the images of the surface cracks before and after healing were almost identical and no healing products were observed. On the contrary, when the CP–S and CM–S specimens containing SAPs were exposed to wet conditions (Figure 9), the crack width decreased due to the formation of healing products within surface cracks during healing. Interestingly, when the CP–S and CM–S specimens were exposed to wet/dry conditions (Figure 10), healing products were not formed and the surface images before and after healing were similar.

**Figure 11:**
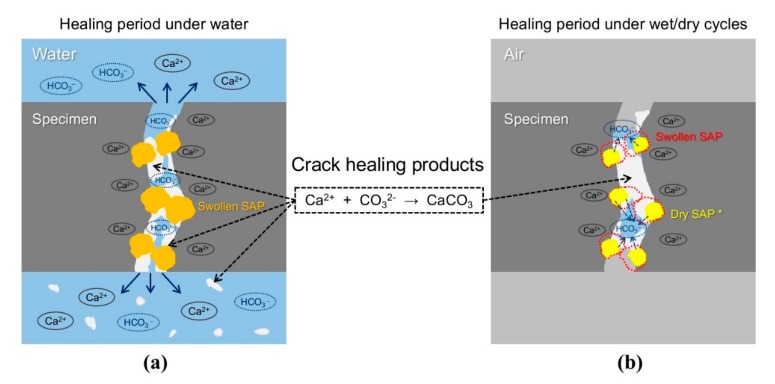
Schematic diagrams showing the mechanisms of autogenous crack healing by SAPs in cracks under (**a**) wet and (**b**) wet/dry conditions.

A schematic diagram showing the mechanisms of crack healing under the different exposure conditions is shown in Figure 11. When the cracked cementitious specimens were exposed to wet conditions (Figure 11a), healing products were more likely to form within internal cracks than external surface cracks. This was because the internal cracks of the specimens offered a favorable environment for precipitating reaction products, as the concentration and pH of ions eluted from the cement matrix increased due to the roughness and bending of the crack surfaces [22]. In addition, when the cracked SAP-containing specimens were exposed to wet conditions, healing products were easily formed around the SAP particles, which remained swollen. Therefore, the precursors of the healing products that could be intensively formed within internal cracks were formed by dispersing around the swollen SAPs inside the cracks. Hence, some healing products were observed in the external surface cracks, as shown in Figure 9. However, as shown in Figure 11b, when the cracked SAP-containing specimens were exposed to wet/dry cyclic conditions, the SAPs swollen by absorbing water during the wet conditions supplied water only to the internal cracks during the dry cycle. Therefore, healing products were intensively formed only inside the cracks due to the internal curing effect of the SAPs; healing products were not observed on the external surface cracks, as shown in Figure 10. In addition, as the swollen SAPs shrank during the drying process, the effective crack width increased as compared to the wet conditions. Therefore, the initial water permeability was temporarily larger than the saturated water permeability, when water was supplied again. In summary, as SAPs absorbed water and swelled, the effective crack width decreased; meanwhile, the water absorbed in the SAPs was released to the crack surfaces under dry conditions, which further augmented autogenous healing, as shown in Figure 11.

Based on image analysis of the surface cracks, the results of the water flow tests of SAP-containing specimens (Figure 6, Figure 7 and Figure 8) are interpreted as follows. The improved autogenous crack healing efficiency of CP–S and CM–S specimens exposed to wet/dry conditions compared to wet conditions was attributed to enhanced formation of healing products in the internal cracks when exposed to wet/dry conditions.

**Figure 12:**
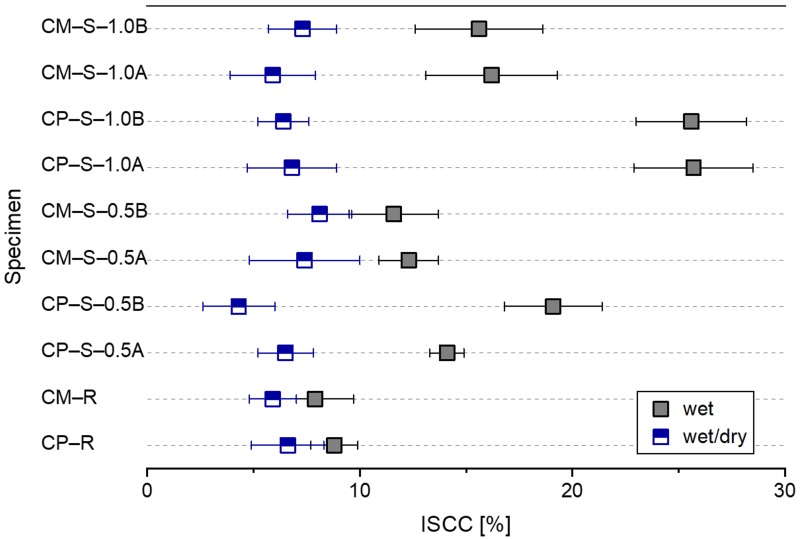
Mean and standard deviation of the index of surface crack closure (ISCC) for all water flow test specimens after healing under wet or wet/dry conditions.

Figure 12 shows the mean and standard deviation of the index of surface crack closure (ISCC) for the different healing conditions. The ISCC was calculated by using Equation (1), as follows:

(1) $$ISCC=\frac{W_{i}-W_{f}}{W_{i}}\times (\%)$$

where *W_i_* is the initial mean surface crack width, and *W_f_* is the final mean surface crack width. Hence, this term quantifies the reduction in surface crack width after healing. In order to calculate the ISCC values shown in Figure 12, *W_i_* and *W_f_* values for the water flow test specimens (data shown in Figure 6) were used. The autogenous crack healing efficiency (Figure 6, Figure 7 and Figure 8) and ISSC (Figure 12) were not proportional to each other. As the cracked cementitious materials exposed to wet conditions more easily formed healing products within the surface cracks, the ISCC values of all specimens for each mixture were larger for wet conditions compared to wet/dry ones. The largest ISCC values (26 ± 3%) were observed for the CP–S–1.0A and CP–S–1.0B specimens. No major differences in the ISCC values were observed for the different SAP particles sizes.

### 3.3. Compressive Strength Recovery

**Figure 13:**
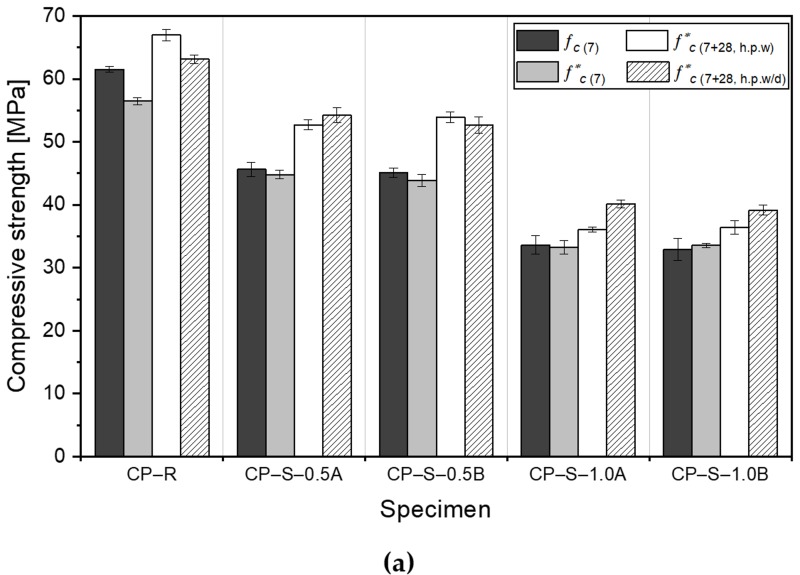

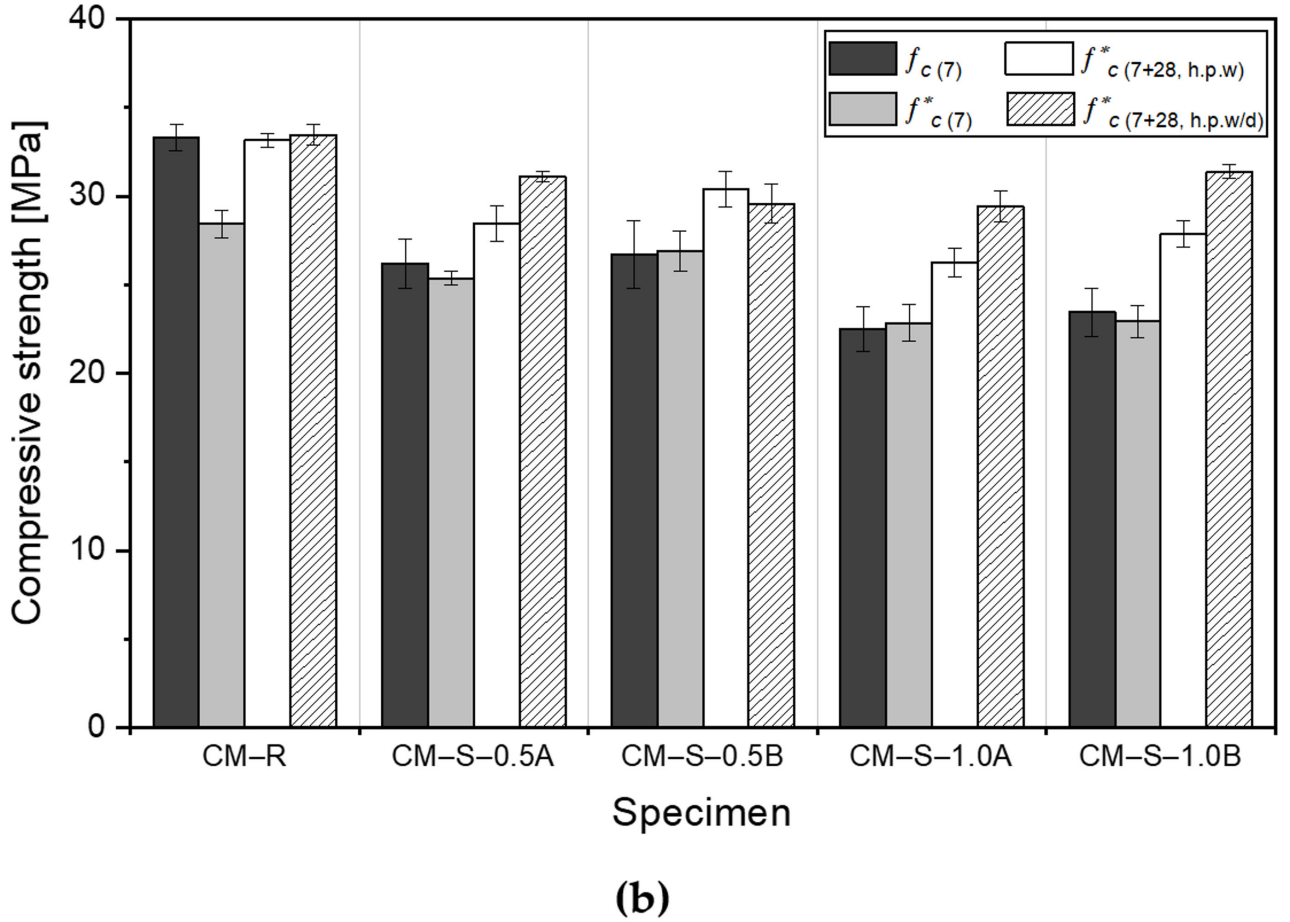
Mean and standard deviation of the compressive strength at 7 d, immediately after pre-cracking, and after the healing period under wet or wet/dry conditions for (**a**) CP and (**b**) CM specimens.

Figure 13 shows the compressive strength of the CP–R, CM–R, CP–S, and CM–S specimens at a curing age of 7 days (*f _c_*_(7)_), immediately after pre-damage (f ^*^_c__(7)_), and after the 28-d healing period under wet (f ^*^_c__(7+28, *h.p.w*)_) or wet/dry (f ^*^_c__(7+28, *h.p.w/d*)_) conditions. The experimental results showed that the addition of SAPs, which acted as defects in the cementitious structure, induced a significant decrease in the *f _c_*_(7)_ values, where the strength decreases with increasing SAP dosage. The SAP particles formed a large number of voids in the cement matrix when they absorbed water and swelled. Hence, more voids were formed with a greater number of SAP particles, resulting in larger strength drops [18,19]. The strength reduction ratio of CP specimens was higher than that of CM specimens for the same SAP dosage. The f ^*^_c__(7)_ values of the CP–R and CM–R specimens decreased slightly compared to the corresponding *f _c_*_(7)_ values, while the CP–S and CM–S specimens showed similar f ^*^_c__(7)_ and *f _c_*_(7)_ values. The CP–R and CM–R specimens had higher initial strength than the CP–S and CM–S specimens due to the dense cement matrix and lack of SAP voids, and were hence more significantly affected by pre-cracking. This was similar to the experimental results of Nardi et al. [39]; they measured the strength of the specimens with/without the self-healing coated granules (which act as defects leading to decrease in strength) at the initial stage and after pre-cracking, where an insignificant difference in the strength was observed for the specimens containing self-healing coated granules.

Figure 13 shows that the compressive strengths of all pre-damaged specimens after the 28-d healing period increased significantly compared to the strength immediately after pre-cracking. This was attributed to the autogenous healing mechanism occurring, where the cracks were filled by the healing products. For the CP–R specimen, f ^*^_c__(7+28, *h.p.w*)_ was greater than f ^*^_c__(7+28, *h.p.w/d*)_ due to the higher degree of autogenous healing and due to the higher availability of water in the wet condition. However, for the CM–R specimen, f ^*^_c__(7+28, *h.p.w*)_ and f ^*^_c__(7+28, *h.p.w/d*)_ were very similar, where the slight difference was attributed only to a slightly different degree of pre-damage. On the contrary, for the SAP-containing specimens, f ^*^_c__(7+28)_ generally increased more when exposed to wet/dry cyclic conditions than when exposed to wet conditions, due to enhanced autogenous healing and due to healing products being intensively formed in the cracks.

Interestingly, however, in the water flow test results shown in Section 3.1, the autogenous healing efficiency of the CP–S–1.0 specimens was superior to that of the CM–S–1.0 specimens. The increase in the compressive strength after pre-cracking of the CP–S–1.0 specimens was expected to be higher than that of the CM–S–1.0 specimens, but the opposite is shown in Figure 13. This may be due to experimental uncertainties related to different initial strengths arising from different internal structures and, hence, different degrees of induced damage during pre-cracking. Therefore, further studies are required for experimental evaluation and causal analysis using a greater number of specimens to improve the statistical results. In addition, the difference in strength due to SAP particle size was minimal and inconsistent (Figure 13), and confirmed to be insignificant.

**Figure 14:**
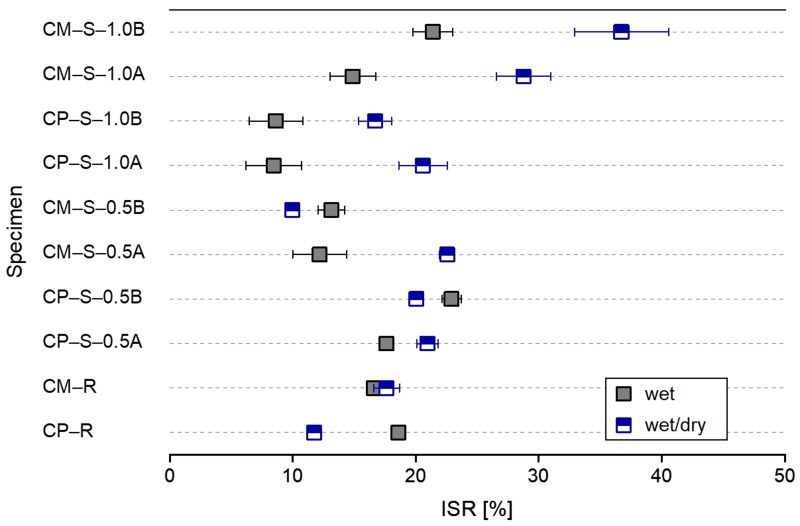
Mean and standard deviation of the index of strength recovery (ISR) after the healing period under wet and wet/dry conditions for all test specimens.

Figure 14 shows the mean and standard deviation of the index of strength recovery (ISR) for the different healing conditions determined by using Equation (2), as follows:

(2) $$ISR=\frac{f^{*}{}_{c(7+28)}-f^{*}{}_{c(7)}}{f^{*}{}_{c(7)}}\times (\%).$$

The ISR was within ~19% for the CP–R and CM–R specimens, 10–23% for the CP–S–0.5 and CM–S–0.5 specimens, and 9–37% for the CP–S–1.0 and CM–S–1.0 specimens. There were differences depending on the exposure conditions and SAP particle sizes. In particular, the ISR for the CM–S–1.0B specimens was 37 ± 4%; this was a very high strength recovery rate.

In addition, considering the compressive strength recovery results, it was expected that the initial flexural strength of SAP-containing specimens would be lower than that of specimens without SAPs, and the degree of flexural strength recovery would increase upon incorporation of SAPs [25].

### 3.4. SEM and FT-IR Results

**Figure 15:**
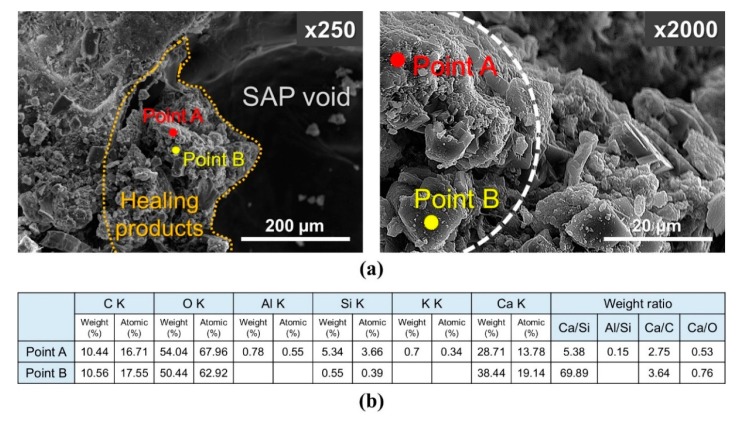
(**a**) SEM micrographs and (**b**) EDS analysis of healing products formed along the surfaces of voids created by swollen SAP particles on the internal crack surface.

As the morphology of healing products formed on the internal crack surface can directly affect the permeability and mechanical properties [40], samples of the healing products were taken from one side of the internal crack surface of an SAP-containing CM specimen upon completion of a 28-d water flow test and analyzed using SEM–EDS. Figure 15a shows SEM images (with various magnifications) that highlight the morphology of the healing products on the surfaces of voids created by SAP particles within the internal crack surface after the test under wet conditions. Figure 15b shows EDS analysis of the healing products from points A and B indicated in Figure 15a. Point A showed mainly Ca, C, and O, along with a small amount of Si and Al, where the weight ratios identified calcium silicate hydrate (CSH) or calcium aluminum silicate hydrate (CASH) as possible hydration products [26]. Point B was mainly composed of Ca, C, and O, with a weight ratio comparable to CaCO_3_ [26]. It is considered that the CSH healing products formed in cracks were the main source of the observed mechanical strength recovery, while the CaCO_3_ healing products had poorer mechanical properties [26]. Therefore, when cracked SAP-containing cementitious materials were exposed to wet conditions, SAP particles in the cracks provided rapid crack self-sealing effects [18] due to their rapid swelling, accompanied by enhanced autogenous crack healing over the long-term by stimulating the formation of healing products, such as CaCO_3_ and CSH, on the surfaces of voids created by the swollen SAP particles.

**Figure 16:**
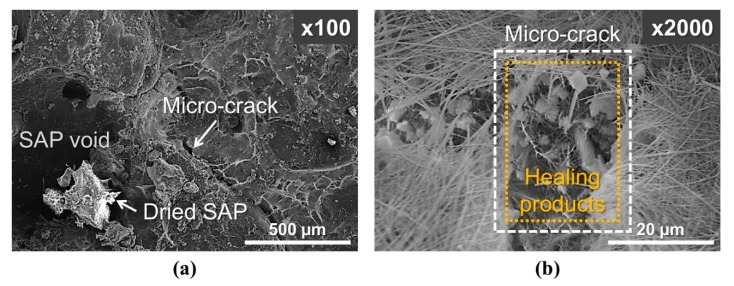
SEM micrographs of the internal crack surface. (a) SAP void with dried SAP particle near micro-crack, (b) healing products formed in the micro-crack near the SAP void.

Figure 16 shows the SEM images of the internal crack surface of an SAP-containing sample after completion of the water flow test under wet/dry conditions. Healing products are clearly visible within the microcrack near the SAP void. As the SAP particles were subjected to repeated swelling/shrinkage under wet/dry conditions, the SAP particles exposed to cracks, which have access to incoming water during wet conditions, repeatedly swelled and supplied water to the nearby crack surface during the dry cycle, improving autogenous crack healing by promoting the formation of healing products. Although other studies have indicated that some SAPs remain physically bonded to the surrounding cement matrix [18] and that there is an interaction between the SAPs and cement that affects the swelling behavior of SAPs [20,41], an analysis is necessary to elucidate the mechanism of the chemical reactions between SAPs and healing products in cementitious materials.

**Figure 17:**
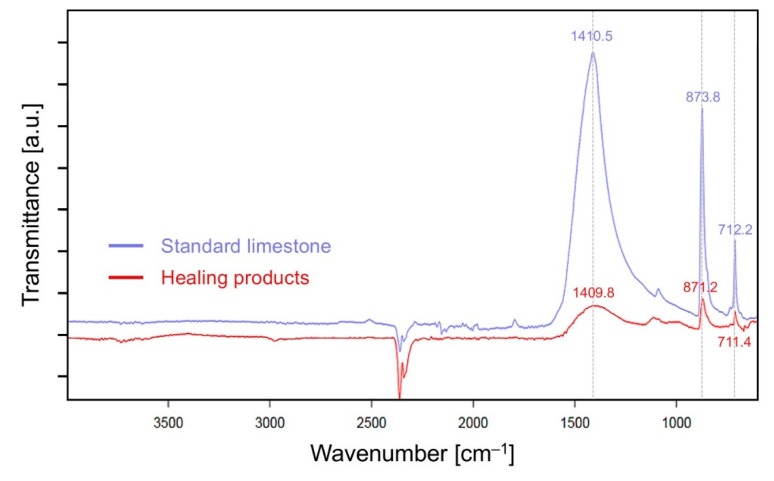
FT-IR spectra for the standard limestone and the healing products obtained after a water flow test of the SAP-containing specimens taken from the external surface cracks.

Figure 17 shows FT-IR spectra of the healing products (white crystalline materials shown in Figure 9) generated on the external surface cracks of the SAP-containing specimens after completion of 28-d water flow tests. The transmittance values do not indicate a quantitative measurement as the sample volume taken from the SAP-containing specimen and that of reference material (standard limestone) used for FT-IR analysis were not equal. The FT-IR peaks of the healing products were in the same locations as those for the standard limestone (consisting of more than 99% CaCO_3_). Hence, the healing products formed on the external surfaces of the specimens were predominantly CaCO_3_ [42], consistent with the findings of Snoeck et al. [25].

## 4. Conclusions

Based on the experimental results obtained from this study, the following major conclusions may be drawn:

- The incorporation of SAPs in cementitious materials with early-age cracking can achieve rapid crack self-sealing in the short term, and also long-term improvements in autogenous crack healing.
- Based on the water flow test results, it is considered that the incorporation of 1.0% SAP can completely block the low-pressure water flowing through cracks over time by approximately less than 300 µm in early-age cementitious materials.
- The flow rates in the first hour of the water flow test varied much more for the CP/CM–S–0.5 and CP/CM–S–1.0 specimens than for the CP/CM–R specimens (without SAPs), as cracks were rapidly sealed due to rapid swelling of the SAPs in the cracks as they absorbed water.
- Water flow tests showed that autogenous crack healing occurred to some extent in the samples without SAPs due to further hydration of the un-hydrated cement particles, although the efficiency of crack healing was greatly improved in the presence of SAPs.
- During the 28-d water flow test, the mean flow rates through the CP–S and CM–S specimens decreased much more than those of the CP–R and CM–R specimens, while the reduction ratio increased with increasing SAP dosage.
- The autogenous crack healing efficiency of the CP–R and CM–R specimens improved when exposed to wet conditions compared to wet/dry conditions, while the opposite was true for CP–S and CM–S specimens.
- When the cracked SAP-containing specimens were exposed to wet/dry conditions, healing products tended to concentrate within the internal cracks.
- Swelling of SAP particles formed multiple voids in the CM, which decreased the initial 7-d compressive strength with increasing SAP dosage. However, the compressive strengths of the pre-cracked SAP-containing specimens after the 28-d healing period increased significantly compared to immediately after pre-cracking. This was attributed to enhanced autogenous healing by the SAPs. The highest ISR was observed for the CM–S–1.0B specimen (37 ± 4%), which is a very high strength recovery rate. This result represents that the mechanical degradation due to the incorporation of SAPs can be solved considerably.
- The major healing products formed by the SAPs were CaCO_3_ and CSH, which were formed along the surface of voids formed by swollen SAPs when immersed in water, and enhanced by wet/dry cyclic conditions due to the internal curing effect of the SAPs.

The results of this study suggest that the SAPs can effectively heal the early-age cracks even when water is supplied intermittently, leading to an increase in extending the service life of concrete.

## Figures and Tables

**Table 1:** Chemical composition of the ordinary Portland cement (OPC) used in this study.

SiO_2_	Al_2_O_3_	Fe_2_O_3_	CaO	MgO	SO_3_	K_2_O	Na_2_O	LOI ^1^
20.5	4.97	3.02	61.8	2.71	2.35	0.72	0.33	2.36

^1^ Loss of ignition.

**Table 2:** Composition of the cementitious mixtures used in this study.

Specimen	d% SAP ^1^ [wt.%]	Cement [kg/m^3^]	Sand [kg/m^3^]	SAP [kg/m^3^]	Water [kg/m^3^]	Water in SAP [kg/m^3^]	SP ^4^ [kg/m^3^]
CP–R	—	1493.7	—	—	522.8	—	—
CP–S–0.5A	0.5 ^2^	1384.1	—	6.9	484.4	69.2	—
CP–S–1.0A	1.0 ^2^	1289.5	—	12.9	451.3	129.0	—
CP–S–0.5B	0.5 ^3^	1384.1	—	6.9	484.4	69.2	—
CP–S–1.0B	1.0 ^3^	1289.5	—	12.9	451.3	129.0	—
CM–R	—	750.4	1275.7	—	262.6	—	7.5
CM–S–0.5A	0.5 ^2^	721.7	1226.9	3.6	252.6	36.1	7.2
CM–S–1.0A	1.0 ^2^	695.1	1181.7	7.0	243.3	69.5	7.0
CM–S–0.5B	0.5 ^3^	721.7	1226.9	3.6	252.6	36.1	7.2
CM–S–1.0B	1.0 ^3^	695.1	1181.7	7.0	243.3	69.5	7.0

^1^ Superabsorbent polymer (SAP) dosage by weight of cement. ^2^ SAP A (particle size: 300–355 µm). ^3^ SAP B (particle size: 425–600 µm). ^4^ Superplasticizer.
